# THE CONNECTION BETWEEN SEROTONIN SIGNALING, AGING, AND IMMUNITY WHEN OBSERVED FROM C. ELEGANS AND HUMAN STUDIES

**DOI:** 10.1093/geroni/igae098.3739

**Published:** 2024-12-31

**Authors:** Liyat Kebbede, Brandy Weathers, Tripti Nair

**Affiliations:** University of Southern California, Leonard Davis School of Gerontology, Los Angeles, California, United States; University of Southern California, Leonard Davis School of Gerontology, Los Angeles, California, United States; University of Southern California, Los Angeles, California, United States

## Abstract

As humans age, various environmental threats impact healthspan. Therefore maintaining an intact immune system is vital. Efficiency of human immune response can decline over time leading to complications with age-related diseases. Homeostatic mechanisms are in place to combat threats to the immune system. Serotonin, a neurotransmitter that critically regulates functions in inflammation and immune response, begins to lack functionality with aging. Signaling dysfunction in neurotransmitters can lead to complications in immune response and negatively impact healthspan. Using C. elegans, the connection between immune response and serotonin signaling can be investigated, as this model organism shares homology with the innate human immune system. The Curran lab designed a mutation in the homeostatic pathway resulting in low serotonin signaling. Consequently, impairment to pathogen sensing occurs. C. elegans rely upon the innate immune system to avoid and survive exposure to harmful bacteria. Their transparent nature and 30 day lifespan, allows for visualization of protein signaling and genetic tracing across the life. With a mutation in SKN-1, it has been observed that they become unaware of the dangers of a lethal pathogen, Pseudomonas aeruginosa (PA14), and are even apathetic towards its threat. The serotonin mutant animals remain on PA14 significantly longer than wild type (WT) animals. This study covers therapeutic interventions with serotonin administration and depletion to observe the neurotransmitters necessity in human clinical trials and C.elegans laboratory studies conducted in the Curran lab. Understanding how serotonin signaling impairs the behaviors of C. elegans underscores its importance in maintaining efficient immune response in humans.

